# Feedback Valence Affects Auditory Perceptual Learning Independently of Feedback Probability

**DOI:** 10.1371/journal.pone.0126412

**Published:** 2015-05-06

**Authors:** Sygal Amitay, David R. Moore, Katharine Molloy, Lorna F. Halliday

**Affiliations:** 1 Medical Research Council—Institute of Hearing Research, Nottingham, United Kingdom; 2 Developmental Science, Division of Psychology and Language Sciences, University College London, London, United Kingdom; Ecole Polytechnique Federale de Lausanne, SWITZERLAND

## Abstract

Previous studies have suggested that negative feedback is more effective in driving learning than positive feedback. We investigated the effect on learning of providing varying amounts of negative and positive feedback while listeners attempted to discriminate between three identical tones; an impossible task that nevertheless produces robust learning. Four feedback conditions were compared during training: 90% positive feedback or 10% negative feedback informed the participants that they were doing equally well, while 10% positive or 90% negative feedback informed them they were doing equally badly. In all conditions the feedback was random in relation to the listeners’ responses (because the task was to discriminate three identical tones), yet both the valence (negative vs. positive) and the probability of feedback (10% vs. 90%) affected learning. Feedback that informed listeners they were doing badly resulted in better post-training performance than feedback that informed them they were doing well, independent of valence. In addition, positive feedback during training resulted in better post-training performance than negative feedback, but only positive feedback indicating listeners were doing badly on the task resulted in learning. As we have previously speculated, feedback that better reflected the difficulty of the task was more effective in driving learning than feedback that suggested performance was better than it should have been given perceived task difficulty. But contrary to expectations, positive feedback was more effective than negative feedback in driving learning. Feedback thus had two separable effects on learning: feedback valence affected motivation on a subjectively difficult task, and learning occurred only when feedback probability reflected the subjective difficulty. To optimize learning, training programs need to take into consideration both feedback valence and probability.

## Introduction

Practice on perceptual tasks in any sensory modality almost always improves performance (e.g., visual: [1]; auditory: [2]; somatosensory: [3]), a phenomenon referred to as ‘perceptual learning’. Feedback on performance, although not always essential for learning [4–7], can facilitate perceptual learning in terms of speed [8], amount of observed improvement [9] and retention of learning over time [10]. However, there is little consensus in the literature regarding how much feedback is useful for optimizing learning, nor on whether it should be given in response to correct or incorrect performance (positive or negative feedback, respectively). This aspect of feedback, often called ‘valence’, has been studied in educational learning contexts [11] as well as in animal learning, and less frequently in human perceptual learning.

Human physiological data suggest that negative and positive feedback are processed in distinct but overlapping networks [12–14], suggesting that they may affect processing and learning in a different manner. Animals tend to learn better under aversive conditions, that is, learning is more effective when an animal is punished for incorrect performance than rewarded for correct performance (e.g., [15]; though see [16]). Human rule-based category learning studies (more similar to the animal learning studies than to perceptual learning) have also found that negative feedback is more effective than positive feedback (see, e.g., [17, 18]). Varying the difficulty of a categorization task with a number of irrelevant dimensions, Meyer and Offenbach [19] concluded that negative feedback was more effective for learning than positive, but only when the task was more difficult (more irrelevant dimensions).

Studies on children in educational settings give a mixed picture on the effects of feedback. On the one hand, positive feedback appears to enhance motivation [20–22]. On the other hand, children appear to learn a discrimination better when incorrect performance is punished (by playing a 98-dB tone) than when correct performance is rewarded (with sweets; [23]). Baumeister et al. [24] suggested that negative feedback is generally more effective than positive because people are more motivated to avoid the negative repercussions of poor performance on their perception of self-efficacy (their perceived ability to influence the outcome). Negative feedback may drive participants to set higher performance goals for their future performance, and thus perform at a higher level than those who receive positive feedback or no feedback at all [25]. Ilgen and Davis [26] found that negative feedback can have two opposite effects: it can spur on greater effort when subjects believe it might improve their performance, but it can also reduce motivation. They therefore concluded that negative feedback may be counterproductive.

These variable and even contradictory results may demonstrate a lack of control of priors: it is not clear whether the punishment and reward provided were equally weighted (subjectively or objectively) in these studies. Nor is it clear whether the positive and negative reinforcement were weighted consistently throughout learning (see [27]): getting another sweet when you only have a few may be more motivating than when you already have many. On the other hand, an annoying tone may retain its aversive nature throughout training. Indeed, there is some evidence that negative and positive feedback may be differently weighted during the time-course of training [28]. Another factor that may influence the relative impact of positive and negative feedback is how much of it is given. Neither the human nor the animal studies controlled for the probability of correct vs. incorrect trials because the feedback accurately reflected performance on the task. Thus, there was no direct control over the weights given positive and negative feedback or reinforcement.

To manipulate feedback independently of task performance, we used a novel paradigm whereby the perceptual discrimination was impossible on all trials [29], and feedback was given randomly in any desired probability [30]. In this discrimination task there are no ‘correct’ or ‘incorrect’ trials, and the feedback does not provide real information about task performance. We have previously utilized this paradigm to demonstrate that the amount of positive feedback needs to be commensurate with the subjectively perceived difficulty of the task in order to facilitate learning [30]. Unlike some previously studied paradigms using biased feedback [9, 31, 32] this manipulation does not promote shifts in the decision criterion without changes in perceptual sensitivity, though concurrent changes in both have also been observed [33]. Using the impossible task, we were able to give equal weight to the negative and positive feedback: 10% negative feedback is the logically same as 90% positive feedback, because on the 90% of trials where no negative feedback was provided listeners could infer they were ‘correct’. Likewise they would be able to infer they were ‘incorrect’ on the 10% of trials where no positive feedback was provided. We were therefore able to examine the effect of feedback valence when the information about ‘correctness’ was equally weighted.

## Materials and Methods

### Participants

Data from 120 adults aged 18–40 are reported here. Seventy participants were recruited for a previous study [30]. Of the remaining 50 participants, 15 were recruited from the student population of University College London, and the remaining 35 from the University of Nottingham student population and from the general public. All participants were paid an inconvenience allowance for their participation. Only participants with normal hearing (pure-tone thresholds bilaterally ≤ 20 dB HL across 0.5–4 kHz) were included in the study.

### Ethics statement

The research protocol was approved by the Nottingham University Hospitals Research Ethics Committee and University College London Research Ethics Committee. Informed written consent was obtained from all participants.

### General procedure

**Fig 1:**
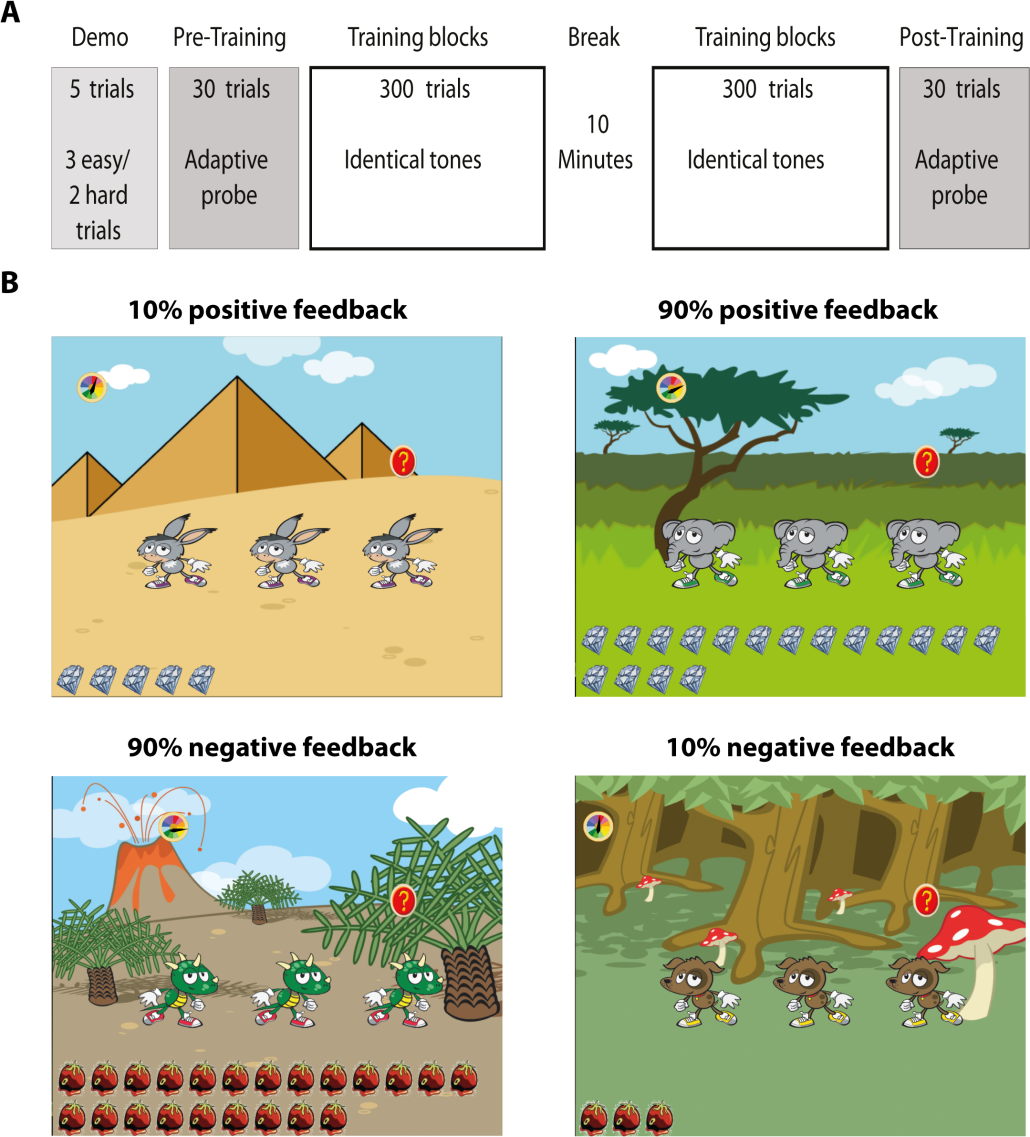
Experimental design. **(A)** The procedure consisted of a pre-test that included a short (5-trial) demonstration to familiarize listeners with the task, followed by a short (30 trials) probe to assess the difference limens for frequency (DLF) [34]. The training phase consisted of 600 trials delivered in 100-trial blocks, with a 10-minute break after the first 3 blocks. Following training, a second probe was administered to assess post-training DLFs. **(B)** Screenshots of the software used for testing and training. Separate groups were trained with 10% and 90% positive feedback (top; [30]), and 10% and 90% negative feedback (bottom). Note that the 10% positive and 90% negative feedback conditions (left column) are equivalent in informing participants that they are doing very badly, while the 90% positive and 10% negative feedback conditions (right column) are equivalent in informing participants they are doing very well.

The study protocol consisted of a pre-test phase, a post-test phase and a training phase (Fig 1A). All testing was completed within one session in a sound-attenuated booth. All phases were administered via “computer games” with a visual interface that cued sound presentation and provided visual feedback during testing and training (Fig 1B). There was no time limit in which to respond, and the initiation of each trial was self-paced.

### Stimuli

Stimuli for both testing and training consisted of three 100 ms tones (including 10 ms raised cosine ramps) presented with an inter-stimulus interval of 500 ms. In the test phases, standard tones had a frequency of 1000 Hz and target tones were varied adaptively. In the training phase, all three tones had a frequency of 1000 Hz. Stimuli were presented diotically using Sennheiser HD-25-1 headphones at 60 dB SPL.

### Pre- and post-training phases

One frequency-discrimination (FD) threshold assessment of 30 trials (‘probe’; see [34]) was administered during each of the pre- and post-training phases. For each trial, listeners were presented with three intervals, two containing the standard tone (F), and the third, randomly-determined interval containing a higher-frequency tone (F + ∆F). Each interval was visually cued by an animated character (see Fig 1B), and listeners were instructed to indicate the interval they believed differed from the other two by touching the character that corresponded to the chosen interval on the touch-screen. Immediate and informative feedback (positive or negative, see below) was received following each trial (see below). A 5-trial demonstration was administered before the pre-test probe to familiarize participants with task requirements. Three of these “demo” trials were ‘easy’ (∆F = 50%), and two were impossible (∆F = 0%). Listeners were instructed to guess when they could not hear a difference between the sounds. All participants correctly identified the target sounds for the ∆F = 50% demo trials.

The probes used an adaptive three-down, one-up staircase procedure, targeting 79.4% correct on the psychometric function [35]. ∆F varied adaptively according to the following rule: starting with ∆F = 50%, it was divided by 2 following every correct response until the first incorrect response. Thereafter, ∆F was divided by √2 after three correct responses, and multiplied by √2 after one incorrect response. Following two consecutive steps in the same direction (up or down), the step size was multiplied by √2. Difference limens for frequency (DLFs) were calculated as the 79.4% correct point on the logistic psychometric function fitted to the 30 trials in each probe using the Wichmann and Hill maximum likelihood procedure [36].

Listeners from the positive feedback groups were allocated based on their pre-test thresholds so as to match the groups as closely as possible on initial FD ability [30]. Listeners from the negative feedback groups were separately matched to the same naïve performance.

### Training phase

During the training phase, all tones were identical (∆F = 0%) but listeners were instructed to perform the same discrimination task as that in the pre-test phase and choose the sound that was different [29]. Although the task is impossible in the sense that there is no right answer (i.e. the tones are all physically identical), subjectively listeners do perceive differences between the stimuli that can be used to make a perceptual judgment (see [37, 38]). Thus the subjective perception is that the task is difficult, but not impossible. Listeners in one training group received positive feedback on 90% of the trials, randomly picked by the software running the experiment. A second training group received positive feedback on 10% of the trials. Two additional groups received negative feedback on 10% and 90% of the trials.

### Feedback

Feedback valence remained consistent throughout testing for each group: groups receiving negative feedback during training also received negative feedback during the pre- and post-test while groups receiving positive feedback also received positive feedback during the pre- and post-test phases. Note that during the pre- and post-test phase, feedback reflected real correct and incorrect responses (for the groups receiving positive and negative feedback, respectively). This was done to avoid confusion on the meaning of the feedback. Feedback valence did not affect pre-test DLFs (see below). Positive feedback for correct responses comprised a brief animation of the correctly chosen character (jumping up and down), explained as a “happy dance”, accompanied by a ‘happy’ sound (‘yippee’) and a diamond token which was added to the cache at the bottom of the test screen (see Fig 1B). Negative feedback included the same brief animation, but this was explained as an “upset gesture” and accompanied by a ‘sad’ sound (a sigh) and a rotten tomato token which was added to the cache. The tokens remained on screen as a cumulative reminder of how ‘well’ or how ‘badly’ participants were doing on the task.

### Data exclusions

Of the 120 participants recruited, 21 were excluded from statistical analysis. As reported in [30] four participants were excluded from the 90% positive feedback group and three from the 10% positive feedback group because the psychometric function fitted to their pre- or post-test probe data had very shallow slopes (≤ 0.10), which rendered the threshold estimates for these probes unreliable. The same criteria were used to exclude 14 listeners allocated to the negative feedback groups following pre-test, with the additional requirement that they matched the range of the original positive feedback group. No additional listeners were excluded based on post-test.

Data analyzed and reported therefore included the original positive feedback cohort (10% positive: n = 31; 90% positive: n = 32), and 36 newly recruited participants (n = 18 each for the 10% and 90% negative feedback groups).

### Statistical analysis

FD threshold data (in Hz) were log-transformed, and all statistical analyses were carried out on the log-transformed data. The learning (pre-test DLFs—post-test DLFs) data met the equality of variance assumption (Levene’s Test of equality of error variance: p = 0.059), and we therefore used a univariate analysis of variance (ANOVA) with between subject factors of feedback valence (positive vs. negative) and feedback meaning (doing well vs. doing badly). We used feedback meaning rather than probability to reflect the information provided by the feedback, where 10% positive feedback was equivalent to 90% negative feedback (indicating listeners are doing badly on the task) and 90% positive feedback was equivalent to 10% negative feedback (indicating listeners are doing well on the task).

## Results

**Fig 2:**
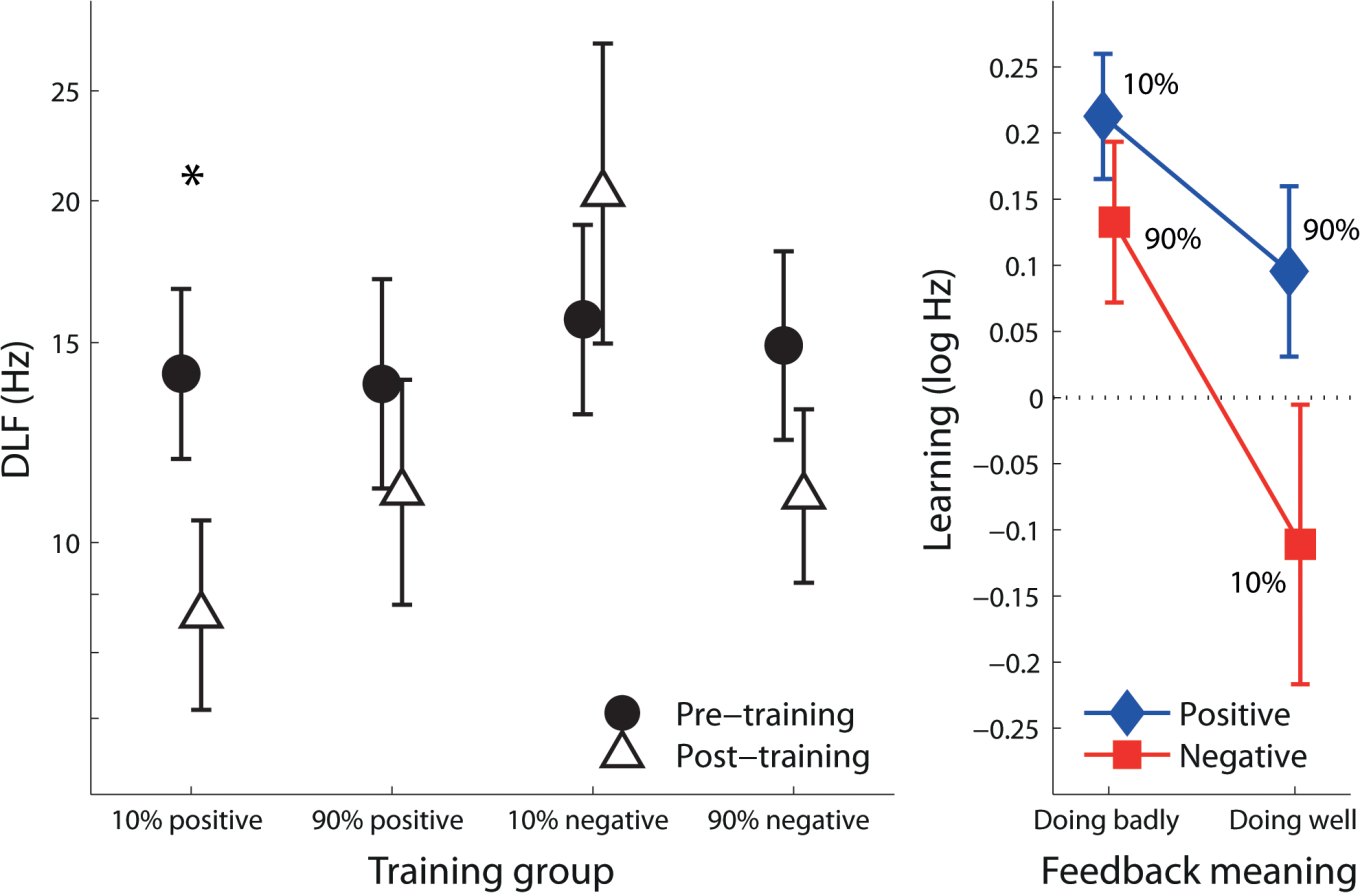
Learning. **(A)** Pre- and Post-training DLFs for each training group. The asterisk demarcates a significant difference between pre- and post-training DLFs (after correction for multiple comparisons). **(B)** Learning—the difference (in logs) between pre- and post-training DLFs for the groups receiving positive and negative feedback. Note that the percentages by the markers indicate the probability of feedback, while the abscissa denotes the meaning conveyed by the feedback. Error bars are s.e.m.

Feedback groups were well-matched on pre-training performance (DLF, Fig 2A; F(3,95) = 0.078; p = 0.97). Positive feedback during training resulted in greater learning than negative feedback (Fig 2B; F(1,95) = 4.15; p = 0.044); feedback that informed listeners they were doing badly was more effective in driving learning than feedback that informed them they were doing well (F(1,95) = 6.58; p = 0.012). Feedback valence did not interact significantly with feedback meaning (F(1,95) = 0.81; p = 0.37).

Of the four training groups, the pre- to post-test improvement was significant only in the 10% positive group (p < 0.001, one-sample t-test, α = 0.0125; Fig 2A). Learning in the 90% negative group was not significant after correction for multiple comparisons (p = 0.044, α = 0.0167). Neither of the groups that received feedback indicating a high success rate on the task showed any improvement.

Although learning varied considerably between listeners in each group, almost all listeners in the groups receiving feedback indicating they were performing poorly improved (or maintained their performance) as a result of training (97% and 89% in the 10% positive and 90% negative feedback groups, respectively; see S1 Fig). On the other hand, a relatively large proportion of those receiving feedback indicating they were performing well showed a decline in performance (28% and 50% in the 90% positive and 10% negative feedback groups, respectively). A χ ^2^ test confirmed that there was a significantly greater proportion of these ‘unlearners’ in the groups receiving feedback indicating they were doing well (χ ^2^ = 6.2; p = 0.013), whereas the difference was not significant when comparing the proportion of unlearners in the negative and positive feedback groups (χ^2^ = 2.0; p = 0.16).

## Discussion

We found that feedback valence and meaning have independent effects on learning: feedback indicating a low success (or high failure) rate was better at driving learning than feedback indicating a high success rate. While positive feedback resulted in better learning than negative feedback, the only condition producing successful learning was training with 10% positive feedback. Perceptual learning on this subjectively difficult task appears to require that feedback should be both realistic and positive.

Based on our earlier study of positive feedback using identical tones [30] we speculated that effective feedback needs to reinforce an internal assessment of performance. However, if this alone accounted for the effects of feedback on learning, our reciprocal negative and positive feedback conditions should have yielded equivalent learning because they were (objectively) equivalent in informing participants of their level of performance on the task. The significant main effect of valence suggests that this was not the whole story.

Valence effects can result from bias. Bias is usually studied with respect to a preference for a specific response over the alternatives (e.g., [39, 40]), but it can also take the form of unequally weighting positive and negative feedback. Thus, the previously observed advantage for negative feedback in both animal [15] and human [21, 41] studies may have resulted from negative feedback being given a greater subjective weight than positive feedback: avoiding punishment for failure may have been given greater importance that receiving a reward for success. The current study suggests that a subjective bias may still exist even when the *objective* “weight” of positive and negative feedback was equated, albeit in the opposite direction to what one would have expected based on the previous literature (e.g., [24]).

The advantage of positive over negative feedback may be related to reward processing in the brain, and specifically to the role of dopamine in both reward processing and plasticity. Unexpected rewards increase the activity of midbrain dopaminergic neurons [42, 43]. Dopamine plays a crucial role in transferring memory traces from short- to long-term memory [44] and supporting learning [45]. A relationship between dopamine, long-term potentiation (a cellular mechanism for synaptic plasticity) and perceptual learning has previously been proposed [46, 47], supported by an imaging study showing better learning for stimuli associated with greater reward [48].

The independent effects of feedback valence and meaning found in the current study may reflect a dissociation between performance-related processing in evaluating feedback based on its probability or meaning and the emotional component associated with its valence [49]. Elliott, Frith and Dolan [49] compared performance on two tasks—one in which participants could control performance (planning) and improve, and one in which they could not (guessing). Unknown to the naïve participants, the feedback in either case was unrelated to performance (as in our study). Yet even when participants became aware of that dissociation over the course of training, feedback valence still influenced their improvement on the planning task. Learning was substantially greater with positive than with negative feedback.

Physiological studies have shown that the brain responds differently to negative and positive feedback [28, 50]. Opitz, Ferdinand and Mecklinger [28] showed that feedback-related positivity (FRP; a positive deflection ERP component elicited by positive feedback) decreased with training, while the feedback-related negativity (FRN; a negative ERP component, elicited by negative feedback) remained constant. Interestingly, recent studies have also found significant individual differences in preference for learning with negative or positive feedback, coined negative and positive learners, respectively [51]. Unger, Heinz and Kray [14] have shown that participants who are sensitive to punishment (i.e. negative feedback) exhibit larger error- and feedback-related negativity (ERN and FRN, respectively), while those more sensitive to reward (positive feedback) showed greater error positivity (Pe). It is possible that our sample was biased towards positive learners, resulting in significant learning only in the 10% positive feedback condition.

The individual variability in our study, reflected in the proportion of participants whose performance declined (“unlearners”) compared to improved (“learners”) from pre- to post-test, was based on feedback meaning rather than its valence. While it might be expected that performance would not improve when the trained task does not promote motivation, a decline in performance is surprising. It is possible that training with feedback indicating good performance, regardless of strategy, led unlearners to stop believing they could influence their own performance (i.e. loss of ‘self-efficacy’; see [26]), and that this effect carried over from the training phase to the post-test, resulting in poor performance. Alternatively, the strategy (or discriminant) these listeners used during the pre-test could have been unlearned due to the exuberant success indicated by feedback during training, leading to poorer post-test performance.

Finally, electrophysiological evidence also suggests that separate ERP components are affected by feedback valence and its probability, and that both components are associated with behavioral adjustment (reviewed in [52]). The amplitude of the P3 ERP component depends on expectations of the outcome: it is larger for unexpected events, including unexpected feedback events, irrespective of feedback valence. This reflects an expectation regarding the probability of correct and incorrect responses, which is likely to be related to the perceived difficulty of the task. Thus, feedback events are treated as good or bad outcomes not in absolute terms but relative to their relationship with expectations, as we have predicted based on previous results from positive-only feedback [30]. These findings may account for the lack of interaction between feedback meaning and valence in the current study.

The manipulation we used here, with random feedback provided on a pre-specified proportion of trials—all of which were identical and impossible to discriminate—allowed us to avoid the pitfalls of previous studies comparing negative and positive feedback as outlined in the introduction. But how generalizable are these findings to learning studies in which feedback accurately reflects performance on the task? It is notable that listeners were unaware that the stimuli within each trial were identical. Subjectively these tones sound different, as confirmed by the debriefing following training—listeners report the task to be “very difficult” but not “impossible”. We have further evidence from electroencephalography (EEG) to support the notion that these tones are perceived as different [38]. Moreover, despite the extreme deviation of feedback from any realistic expectation (10% is significantly below chance level on a 3-alternative, forced-choice task), among the many listeners already tested with this impossible task (e.g., [30]) only one reported noticing that feedback was less than it should be by chance in the 10% positive condition (this listener was not part of the experiment reported here). We thus suggest that the effect of feedback probability, at least, is generalizable to more realistic probabilities (usually 70–80% correct in psychophysical studies). We suggest that so long as the feedback probability, or the information it conveys about performance, does not significantly diverge from the internal assessment of task difficulty it should drive learning. Hence, these results should be generalizable to studies where differences between both stimuli and feedback are ‘real’. The results should also be generalizable to other perceptual learning tasks—we have no reason to think that the mechanisms responsible for processing the performance-related and emotional aspects of feedback are specific to frequency discrimination, or that they are specific to the way learning is driven in a frequency discrimination task.

To conclude, the current study suggests that positive is superior to negative feedback in driving learning, and that the feedback needs to reflect the difficulty of the task—learning does not occur when the feedback informs learners that they are doing better than they should be given the task’s difficulty.

## Supporting Information

S1 Fig Individual difference limens for frequency (DLFs) before and after training. Data are shown for pre- and post-test for each of the four training groups. The top two panels are for the groups receiving positive feedback and the bottom two panels for those receiving negative feedback. The two panels on the right show listeners for whom the feedback indicated they were doing well, while the panels on the left show listeners for whom the feedback indicated they were doing badly.
(TIF)
