# Supplementary figures and images for: Feedback Valence Affects Auditory Perceptual Learning Independently of Feedback Probability
Source: PLoS One. 2015 May 6;10(5):e0126412. doi: 10.1371/journal.pone.0126412 (PMC4422442; doi:10.1371/journal.pone.0126412)

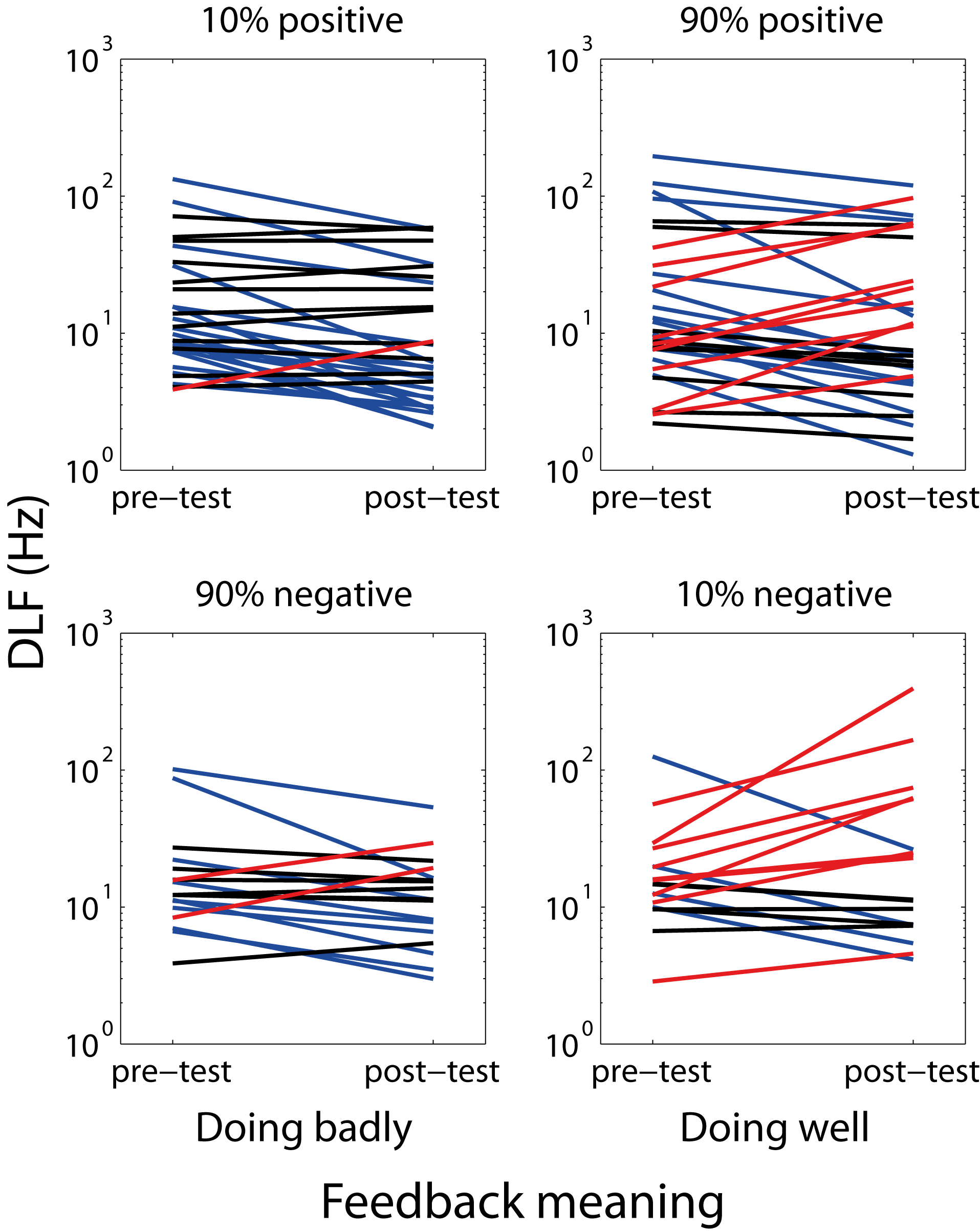

Supplement: S1 Fig — Data are shown for pre- and post-test for each of the four training groups. The top two panels are for the groups receiving positive feedback and the bottom two panels for those receiving negative feedback. The two panels on the right show listeners for whom the feedback indicated they were doing well, while the panels on the left show listeners for whom the feedback indicated they were doing badly. (TIF) [file pone.0126412.s001.tif]
